# From Community Laywomen to Breast Health Workers: A Pilot Training Model to Implement Clinical Breast Exam Screening in Malawi

**DOI:** 10.1371/journal.pone.0151389

**Published:** 2016-03-09

**Authors:** Lily Gutnik, Agnes Moses, Christopher Stanley, Tapiwa Tembo, Clara Lee, Satish Gopal

**Affiliations:** 1 UNC Project-Malawi, Lilongwe, Malawi; 2 Department of Surgery, Montefiore Medical Center/Albert Einstein College of Medicine, Bronx, New York, United States of America; 3 Lineberger Comprehensive Cancer Center, University of North Carolina, Chapel Hill, United States of America; 4 Department of Surgery, University of North Carolina, Chapel Hill, United States of America; 5 Institute for Global Health and Infectious Diseases, University of North Carolina, Chapel Hill, United States of America; 6 University of Malawi College of Medicine, Blantyre, Malawi; Iranian Institute for Health Sciences Research, ACECR, ISLAMIC REPUBLIC OF IRAN

## Abstract

**Background:**

Breast cancer burden is high in low-income countries. Inadequate early detection contributes to late diagnosis and increased mortality. We describe the training program for Malawi’s first clinical breast exam (CBE) screening effort.

**Methods:**

Laywomen were recruited as Breast Health Workers (BHWs) with the help of local staff and breast cancer advocates. The four-week training consisted of lectures, online modules, role-playing, case discussions, CBE using simulators and patients, and practice presentations. Ministry of Health trainers taught health communication, promotion, and education skills. Breast cancer survivors shared their experiences. Clinicians taught breast cancer epidemiology, prevention, detection, and clinical care. Clinicians and research staff taught research ethics, informed consent, data collection, and professionalism. Breast cancer knowledge was measured using pre- and post-training surveys. Concordance between BHW and clinician CBE was assessed. Breast cancer talks by BHW were evaluated on a 5-point scale in 22 areas by 3 judges.

**Results:**

We interviewed 12 women, and 4 were selected as BHWs including 1 breast cancer survivor. Training was dynamic with modification based on trainee response and progress. A higher-than-anticipated level of comprehension and interest led to inclusion of additional topics like breast reconstruction. Pre-training knowledge increased from 49% to 91% correct (p<0.0001). Clinician and BHW CBE had 88% concordance (kappa 0.43). The mean rating of BHW educational talks was 4.4 (standard deviation 0.7).

**Conclusions:**

Malawian laywomen successfully completed training and demonstrated competency to conduct CBE and deliver breast cancer educational talks. Knowledge increased after training, and concordance was high between BHW and clinician CBE.

## Introduction

Breast cancer burden has increased more rapidly in low and middle income countries (LMIC) than in the developed world, and 51% of global breast cancer cases now occur in LMIC [1,2]. In Malawi, breast cancer is the third most common cancer among women [2]. Women in LMIC, including Malawi, often are diagnosed with late stage disease that is more difficult to treat and less amenable to cure [3,4]. Consequently, earlier breast cancer detection is needed in LMIC [4]. Although screening mammography identifies earlier-stage cancers in resource-rich settings, it is typically unavailable in developing countries, which often lack the necessary infrastructure and personnel to implement it [5]. As a result, other screening approaches such as clinical breast exam (CBE) may be more appropriate in LMIC [6,7].

CBE has shown potential as an early detection method in LMIC to identify breast cancer at earlier stages among symptomatic women and as a screening intervention applied to asymptomatic women [8–13]. Community health workers and laywomen have been able to effectively perform CBE, achieving high concordance with physician CBE. CBE screening using this approach has resulted in a higher proportion of early stage tumors compared to self-detected cancers [10–14]. In LMIC settings with severe human resource limitations, task shifting medical care to lower cadres of health workers, including trained volunteers, has been a key element in successful public health interventions such as antiretroviral therapy scale-up and maternal and child health [15–17]. Similar approaches might be applied to cancer screening. Although adequate training and quality assurance are critical to the success of such cancer screening programs, few descriptions of the implementation aspects of such programs exist.

To address these issues, we conducted a pilot study, training laywomen to become breast health workers (BHWs) in Malawi. BHWs were taught to perform CBE and give standardized breast cancer talks. In this paper, we describe implementation of our BHW training model in the capital, Lilongwe. Results of the pilot CBE implementation study, which occurred subsequent to BHW training, are not included here and will be reported separately.

## Methods

### Recruitment and Logistics

Job descriptions were posted at UNC Project, the local study site, and were distributed to the breast cancer advocacy community of Lilongwe. The principal investigator interviewed a total of 12 women. Among these women, six were selected for a second interview with two senior co-investigators. Four women were finally selected, including one breast cancer survivor, all or whom were from the local communities of Lilongwe. After recruitment, training was conducted over four weeks, with BHWs attending five days per week for eight hours each day. Training took place in conference rooms and lectures halls of UNC Project, and the training period was part of the BHW salaried contract.

### Curriculum Development

#### Health Surveillance Assistant Training

Akin to the role of a community health worker, Malawi employs Health Surveillance Assistants (HSAs). To introduce BHWs to the health sector, modules from the HSA curriculum (primary care, essential health package, patient follow up, health education and promotion, interpersonal communication and counseling skills, and female reproductive health system) were taught via lectures and role-playing by Ministry of Health HSA trainers during the first week of training. Paired with HSAs, BHWs also practiced delivering educational health talks in clinics.

#### Breast Cancer Knowledge

We created the BHW training manual and residential course based on breast cancer patient information sources and images from the U.S. National Cancer Institute, American Cancer Society, Susan G. Komen Foundation, Jhpiego, WHO, and Worldwide Breast Cancer. These patient information resources were specifically chosen based on appropriate language and comprehension level for laywomen. The following topics were included: breast cancer epidemiology, breast anatomy and physiology, common benign breast conditions, breast cancer causes, types of breast cancer, risk factors, screening, diagnosis, and treatment. The pre- and post-test was adapted from a Mexican breast cancer training intervention utilizing community health workers[18]. Three investigators conducted the didactic sessions over approximately 7 days. Results of the pre-test were shared with the BHWs throughout the training as relevant topics were taught. The pre-test questions were used to initiate training discussions. Each topic had a didactic lecture followed by multiple-choice questions that were discussed as a group. The multiple-choice questions were chosen from the book *Breast Care*, a breast course curriculum for health professionals developed in South Africa [19]. At the end of the training, BHWs completed the post-test survey.

#### Breast Cancer Attitudes and Beliefs

The breast cancer-specific aspects of training commenced with an in-depth and personal discussion of breast cancer in Malawi. Two breast cancer survivors, including one BHW, shared their stories. The second survivor is Malawi’s leading breast cancer advocate and a former Miss Malawi diagnosed with breast cancer at a young age. Their personal accounts helped to reduce the stigma associated with the disease. The most common obstacles to breast cancer care in Malawi, as perceived by BHWs, were lack of knowledge and awareness, financial hardships, and the concept that cancer diagnosis equals death. These themes were used to guide the training, and efforts were made to specifically correct common misconceptions. This discussion took place for approximately one-half day. Lectures about treatment went beyond clinical aspects and focused on the experience of Malawian cancer patients, including costs and wait times for diagnosis and treatment.

#### Clinical Breast Exam

A 15-minute video on CBE technique was shown followed by a didactic lecture of each step [20]. Subsequently, the technique was demonstrated on the Nasco Life/Form Advanced Breast Exam Simulator®. BHWs were also taught to obtain a breast health history. They were then taught to visually inspect breasts for asymmetry, visible lumps, skin changes, edema, nipple retraction, discharge or axillary swellings while the woman is in an upright position with hands on her hips and in a supine position. To palpate the breast, BHWs were taught to use the pads of the middle three fingers with overlapping dime-sized circular movements while the woman is in a supine position with the ipsilateral arm overhead to flatten palpation of axillary. Axillary and supraclavicular lymph node palpation were taught while the woman is in upright position. BHWs completed simulated exams followed by examination of patient volunteers recruited from UNC Project clinics, accompanied by study physicians. After each exam, there was a group debriefing beginning with self-evaluation by each BHW, then feedback for each other, and lastly physician feedback. Learning and practicing CBE took approximately 7 days.

#### Breast Cancer Educational Talks

BHWs learned a standardized breast cancer talk utilizing a flip chart adapted from a health promoter intervention in Mexico [18]. Each BHW talk was given to peers and UNC Project clinical research colleagues and evaluated using an observation instrument from the University of Arizona CHW Evaluation Toolkit by three independent judges who were Malawian project staff unaffiliated with the study [21]. Time dedicated to learning, practicing, and testing the talks was approximately three days.

#### Professionalism

Medical professionalism was taught via a short lecture followed by 30 practice scenarios. The scenario topics included clinical staff encounters, patient-related concerns, emergencies, confidentiality issues, space issues, and study integrity. For example, one scenario was about a patient suddenly becoming severely short of breath during CBE. BHWs worked in pairs to discuss how to best handle each scenario, followed by a larger group discussion.

#### Research Training

In the main study, BHWs conducted CBE and delivered breast cancer education talks, but also recruited and enrolled study participants, obtained informed consent, and collected study data. To prepare for their role as CBE examiners and research assistants for a CBE implementation study in Lilongwe that was conducted after the training program, BHWs completed the CITI online program courses on Good Clinical Practice (GCP) and Human Subjects Research at UNC Project computer stations monitored by local staff during business hours. The purpose of the training was to equip them with knowledge on research ethics. Project regulatory staff led live sessions on obtaining informed consent from study participants. Subsequently, BHWs practiced the informed consent process with female employees. Data collection forms were explained with the help of several practice scenarios. Each BHW practiced the entire study process from educational talk delivery to recruitment to actual CBE including completion of all data instruments. Experienced project research assistants observed the process and provided advice and feedback. Finally, all study sites were visited to allow BHWs to become acquainted with local staff, clinic workflow, and study space.

### Data Collection

#### Training Evaluation

We developed a 14 question post-training survey. Ten questions used a Likert-scale format and were centered on the trainers, the environment, and the content. One question asked the overall rating of the training. Three free-text questions asked BHWs to cite training strengths and weaknesses, and to provide other comments.

#### Data Analysis

Pre- and post-training knowledge scores were compared with test of proportions. Kappa concordance was calculated for BHW and physician exam. The breast cancer talk evaluation instrument uses a 5-point scale for 22 individual topics in four main areas: introduction, delivery and presentation skills, knowledge of subject, and interaction with participants. There was also space for additional comments.Mean scores with standard deviations of the breast cancer talk evaluations were calculated for each main area and overall. Analyses were performed using STATA SE version 12.0 (College Station, Texas).

#### Ethical Approval

The study was approved by the Biomedical Institutional Review Board of the University of North Carolina at Chapel Hill, the Protocol Review Committee of the Lineberger Comprehensive Cancer Center, and the Malawi National Health Sciences Research and Ethics Committee. After study implementation, women undergoing CBE provided written informed consent using informed consent forms approved by the ethics committees.

## Results

### BHW Traits

Table 1 shows the characteristics of the BHWs. The median age was 31.5 years (range 22–58). All had at least secondary school education, and all had some prior breast cancer knowledge. Friends were the main source of breast cancer knowledge shared by all BHWs. We only considered female BHW candidates due to cultural concerns regarding CBE conducted by laymen.

**Table 1:** Characteristics of Breast Health Workers.

BHW	Age	Marital Status	Highest Educational Attainment	Most Recent Work Experience	Sources of Acquired Breast Cancer Information
**1**	22	Single	College	Assistant primary school teacher	Friends, Flyers, Internet
**2**	27	Married	Secretarial school, certificate in information technology	Administrative assistant in private hospital	Friends, School, Magazines, Newspapers, Internet,roomate who had and died of breast cancer
**3**	36	Married	Teacher’s college and certification	Completed teaching certification	Family, Friends, School, Radio, TV, Health Center,Magazines
**4**	58	Married	Secretarial school	Corporate administrative assistant	Family, Friends, Magazines, Personal experience

### Breast Cancer Knowledge

Pre- and post-test surveys showed global improvements in knowledge (Table 2). Collectively, pre-training knowledge increased from 49% to 91% correct (p<0.0001). The biggest domains of knowledge improvement were causes of breast cancer, risk factors, signs and symptoms, and treatment. BHW comprehension and interest led us to include these additional topics: breast reconstruction, palliative care, and survivorship. We also pursued greater depth regarding breast cancer epidemiology, subtypes, and imaging. BHWs also requested to have their own discussion groups in Chichewa after each English lecture and quiz. This helped solidify understanding, as they raised more questions and asked for further clarification from study investigators for particular issues in English after discussion amongst themselves.

**Table 2:** Pre- and post-training survey results for Breast Health Workers.

Breast Cancer Content Area (# of questions)	BHW 1% correct		BHW 2% correct		BHW 3% correct		BHW 4% correct		All % correct	
	Pre	Post	Pre	Post	Pre	Post	Pre	Post	Pre	Post
**Risk Factors (12)**	25	100	8	92	17	75	58	92	**27**	**90**
**Signs and Symptoms (12)**	42	92	58	92	83	92	75	92	**71**	**92**
**Epidemiology (3)**	100	100	100	100	100	100	100	100	**100**	**100**
**Treatment (6)**	83	83	50	83	33	83	67	83	**46**	**83**
**Defining Family History (9)**	89	89	78	100	89	89	89	78	**86**	**89**
**Clinical Breast Exam (6)**	33	100	33	83	50	100	33	100	**38**	**96**

BHW = Breast Health Worker.

### Breast Cancer Educational Talks

The mean rating of BHW educational talks was 4.4 (standard deviation 0.7) out of 5. The BHWs also received constructive feedback in free text comments to help them deliver more effective messages in clinical environments. Examples of such feedback included “good communication skills, easy to educate locals about the disease” and “she needs to relax, talk slowly a bit, she's too fast. People have different IQs so if she's too fast they may not get what the study is about.” Another comment was “should add some engagement while asking questions or add some jokes.”

### CBE

Introducing CBE with an instructional video proved to be valuable, as it allowed participants to notice nuances and differences in technique when CBE was taught using the simulator. After 12 total practice rounds on the simulator, two BHWs volunteered to have the others practice CBE on them to have a sense of what real breast tissue is like. Subsequently, the BHWs examined 35 patients supervised by a physician investigator. Each BHW exam was followed by a physician exam on the same patient. The patient was always asked how the BHW exam compared to the physician exam to assess the examinee’s general experience. One patient commented, “She [BHW] is very well trained because the exam felt exactly like the doctor.” Physician and BHW CBE had 88% concordance (kappa = 0.43), indicating moderate to good agreement during training. Two out of four discordant exams involved abnormal BHW exams followed by normal clinician exams.

### Research Training

All BHWs successfully completed online modules and obtained research ethics certificates. In the training evaluation, one BHW stated: “the online training also helped us to understand why it is necessary to explain to the client the consent form before exam.” We revised all of the study data instruments based on BHW feedback. Practicing the entire process from giving the educational talk, performing CBE, and collecting data familiarized BHWs with the anticipated workflow and allowed them to provide input into study processes. For example, experienced RAs assisted BHWs in learning how to obtain thoroughly informed consent efficiently in busy clinical environments.

### Training Evaluation

The overall mean rating of the training was 4.75 (standard deviation 0.5) out of 5. Comments from independent assessors were predominantly positive such as, “everything was explained clearly, and we were also able to ask questions and even participate in the amendments of different changes.” One BHW thought that the HSA training was too long. Another BHW disliked the simulator and preferred to learn CBE on women.

## Discussion

Our pilot training model allowed four laywomen to develop competency to deliver accurate breast cancer knowledge, educate fellow women, conduct clinical breast exams, recruit and enroll participants, and collect research data. BHW breast cancer knowledge improved significantly after the training, which addressed common breast cancer misconceptions among Malawian women. BHW breast cancer education talks were also highly rated by their peers, and survey responses demonstrated high levels of satisfaction with the training overall. After training, they participated in an urban CBE screening implementation study of 1000 women attending Lilongwe health clinics, results of which will be separately reported.

The success of our pilot training model may be largely due to our BHW selection process. The job description specifically focused on candidates with a stated passion and commitment to improve breast cancer awareness and early detection in Malawi. All but one BHW had a personal connection to breast cancer, and all of them expressed a deep commitment to the advancement of Malawian women. Regarding the training itself, an introduction to health care in Malawi by experienced HSA trainers eased BHWs into the clinical environment before then focusing on breast cancer and program objectives. Open discussions about common challenges and taboos regarding breast cancer in Malawi, including strong narratives of successful breast cancer diagnosis and treatment by survivors, emphasized the importance of the BHWs. BHWs appreciated supervised training with actual patients and expressed satisfaction with learning something new from each individual patient. The professionalism scenarios stimulated thoughtful discussion and provided concrete strategies for dealing with challenging situations in the clinical environment. Finally, throughout the training, BHW feedback was constantly used to adapt and tailor the curriculum to maximize their learning. This helped build rapport between BHWs and study investigators.

The research training component proved to be most challenging and took the longest time to successfully complete. The research content was strategically placed at the end of the training, but comprehension may have been hindered by BHW fatigue at that stage. Role- playing and dry runs were the most helpful in familiarizing BHWs with data instruments and procedures. BHWs found performing CBE and health talks satisfying and manageable, but initially found it difficult to keep up with data collection in clinic. Malawian clinical environments are often chaotic despite efforts to provide structure. Based on BHW feedback, we condensed and simplified data instruments resulting in more successful and systematic data collection.

This training program provides one informative model for potential scale-up elsewhere in Malawi and other low-resource settings. To our knowledge, there are no detailed published descriptions of training laywomen to perform CBE and educate the community on breast cancer in LMIC. Although we did not conduct formal cost effective analyses, our model did not require costly supplies or advanced technology. The most expensive training tool was the breast simulator, but BHWs preferred to practice on actual women (including each other as well as patients). The estimated total costs for the four-week training were approximately 2000 USD, not including investigator/trainer time. There are many free breast cancer resources aimed at patients and the lay community which are publicly available online and in print from various organizations. Selections from these sources were successfully merged together and adapted to build a comprehensive curriculum that seemed to meet local needs and training objectives.

Our training program required a substantial commitment of time by BHWs and trainers. However, if similar approaches were adopted elsewhere, some aspects of our curriculum may not be required. For example, research training might be omitted in other programs, but was necessary for our BHWs because they also served as research assistants who obtained informed consent and collected research data. Likewise, our BHWs had no prior training as community health workers, but for cadres with these skills, breast cancer-specific aspects of the training could suffice without other modules. Notably, the modular nature of our training program should lend itself well to adoption of relevant sections in other settings depending on local needs and objectives.

We found that openly addressing local cultural issues including prevalent myths and taboos from the beginning was very important. Likewise, we found it highly beneficial to engage the local breast cancer advocacy community in identifying candidates for BHW positions and implementing the program.

We trained only four women, which created a learning environment conducive to curriculum customization and adjustment of pace based on comprehension and interests, perhaps contributing to very high satisfaction on the training survey. This may be more challenging with larger groups of women, and based on our experience, we would recommend frequent small group discussions and role- play exercises embedded within larger trainings. Additionally, these four urban women were highly motivated and all had post-secondary education. It is unclear if this model would work as well with rural or less educated women acting as BHWs, which is an important caveat with respect to the scalability and generalizability of our program to other settings. Finally, breast cancer knowledge and satisfaction scores with training were assessed using instruments that have not been extensively validated in the Malawian population, as such instruments do not currently exist to our knowledge.

Our pilot training model demonstrates that appropriately selected laywomen can be an important group to engage and leverage for breast cancer awareness and early detection in resource-limited settings. Although we drew on numerous publicly available educational sources to develop our curriculum, there is little guidance regarding what content to include and how to train laywomen effectively to perform CBE and promote breast cancer awareness in comparable settings. Further research into curricular optimization and standardization for lower cadres of health workers and/or laypersons can facilitate expansion of similar programs in LMIC settings, where more highly skilled health care workers are scarce. We hope our model can provide some direction to other programs in similar environments, and will report more mature clinical and programmatic outcomes for the CBE screening intervention subsequently.
